# Are humans the initial source of canine mange?

**DOI:** 10.1186/s13071-016-1456-y

**Published:** 2016-03-25

**Authors:** Valérie Andriantsoanirina, Fang Fang, Frédéric Ariey, Arezki Izri, Françoise Foulet, Françoise Botterel, Charlotte Bernigaud, Olivier Chosidow, Weiyi Huang, Jacques Guillot, Rémy Durand

**Affiliations:** Parasitology- Mycology Department, Avicenne Hospital, AP-HP, Bobigny, France; Parasitology Department, College of Animal Science and Technology, Guangxi University, Nanning, China; Research group Dynamyc, EnvA, UPEC, Maisons-Alfort & Créteil, Paris, France; Parasitology- Mycology Department, Cochin Hospital, AP-HP, Inserm U1016, Université Paris Descartes, Paris, France; UMR 190, Unité des virus émergents, Université Aix-Marseille, Faculté de Médecine-Timone, Marseille, France; UFR SMBH, Université Paris 13, Bobigny, France; Parasitology- Mycology Department, Henri Mondor Hospital, AP-HP, Créteil, France; Dermatology Department, Henri Mondor Hospital, AP-HP, UPEC, Créteil, France; UMR216, Mère et enfant face aux infections tropicales, Faculté des Sciences Pharmaceutiques et Biologiques, Université Paris Descartes, Paris, France

**Keywords:** *Sarcoptes scabiei*, Scabies, Sarcoptic mange, Humans, Dogs, Canids, Host switch, Phylogenetic analysis

## Abstract

**Background:**

Scabies, or mange as it is called in animals, is an ectoparasitic contagious infestation caused by the mite *Sarcoptes scabiei.* Sarcoptic mange is an important veterinary disease leading to significant morbidity and mortality in wild and domestic animals. A widely accepted hypothesis, though never substantiated by factual data, suggests that humans were the initial source of the animal contamination. In this study we performed phylogenetic analyses of populations of *S. scabiei* from humans and from canids to validate or not the hypothesis of a human origin of the mites infecting domestic dogs.

**Methods:**

Mites from dogs and foxes were obtained from three French sites and from other countries. A part of cytochrome *c* oxidase subunit 1 (*cox*1) gene was amplified and directly sequenced. Other sequences corresponding to mites from humans, raccoon dogs, foxes, jackal and dogs from various geographical areas were retrieved from GenBank. Phylogenetic analyses were performed using the *Otodectes cynotis cox*1 sequence as outgroup. Maximum Likelihood and Bayesian Inference analysis approaches were used. To visualize the relationship between the haplotypes, a median joining haplotype network was constructed using Network v4.6 according to host.

**Results:**

Twenty-one haplotypes were observed among mites collected from five different host species, including humans and canids from nine geographical areas. The phylogenetic trees based on Maximum Likelihood and Bayesian Inference analyses showed similar topologies with few differences in node support values. The results were not consistent with a human origin of *S. scabiei* mites in dogs and, on the contrary, did not exclude the opposite hypothesis of a host switch from dogs to humans.

**Conclusions:**

Phylogenetic relatedness may have an impact in terms of epidemiological control strategy. Our results and other recent studies suggest to re-evaluate the level of transmission between domestic dogs and humans.

## Background

Scabies, or mange as it is called in animals, is an ectoparasitic contagious infestation caused by the mite *Sarcoptes scabiei* [1–4]. This neglected and emerging/re-emerging disease is a significant public health problem worldwide with an estimated number of cases in humans of over 100 million in 2010 [5]. Sarcoptic mange is also an important veterinary disease leading to significant morbidity and mortality in wild and domestic animals. It affects more than 100 species of mammals worldwide including companion, livestock, and wild animals and it is an emerging problem in many countries [3, 6]. For many years, host-associated populations of *S. scabiei* have been taxonomically divided into morphologically indistinguishable varieties [3, 7, 8]. The host-specificity of these varieties is still controversial, and current studies are investigating whether they belong or not to different species. Cross-infectivity was observed experimentally on some occasions [4, 9, 10]. Natural apparent cross-infectivity has been recently reported in sympatric wild animal host populations [11–14]. Transmission of scabies mites between other species and humans are common, usually leading to clinically moderate and self-limiting forms, though they may persist for several weeks or in rare cases, until treated [7, 15–20]. In particular, the domestic dog is reportedly the most frequent non human reservoir of mites infecting humans, which may have some implications in term of transmission and control of scabies [21–24].

A widely accepted hypothesis, though never substantiated by factual data, suggests that humans and protohumans were the initial source of animal contamination, dogs and other domestic animals being infested by human contacts and themselves a source for other species of wildlife [3, 4, 7, 25]. In this study we performed phylogenetic analyses of populations of *S. scabiei* in humans and in canids to validate or not the hypothesis of a human origin of the mites infecting domestic dogs.

## Methods

### Ethical approval

Mites from humans included in this work were obtained in a study reviewed and approved by the *Comité de Protection des Personnes* (institutional review board) of the ethic committee CPP-Ile-de-France X (approval# 2012/10/23); informed consent was obtained from all patients.

### Collection of *S. scabiei* mites

Mites from dogs and foxes (*Vulpes vulpes*) were obtained from the collection of the Parasitology Department of the Veterinary College of Alfort, Maisons-Alfort, France and two other French sites, and from other countries (Table 1). All cases were independent; only one mite per different dog was included in the study.

**Table 1:** List of *Sarcoptes scabiei* sequences used in this study

Haplotype	Sample name	Host	Scientific name	Location	GenBank reference	Reference
1	canis10	Dog	*Canis lupus familiaris*	Australia	AY493391	[35]
2	canis202	Dog	*Canis lupus familiaris*	Australia	AY493392	[35]
3	canis22	Dog	*Canis lupus familiaris*	USA	AY493393	[35]
3	Sc38	Raccoon dog	*Nyctereutes procyonoides*	Japan	AB821008	[12]
3	Sc24	Raccoon dog	*Nyctereutes procyonoides*	Japan	AB821006	[12]
3	Sc20	Raccoon dog	*Nyctereutes procyonoides*	Japan	AB821005	[12]
3	S16	Human	*Homo sapiens*	France	-^a^	[32]
3	dog3_china	Dog	*Canis lupus familiaris*	China	KT961022	This study
3	dog_italy	Dog	*Canis lupus familiaris*	Italy	KT961025	This study
3	dog1_france	Dog	*Canis lupus familiaris*	France	KT961029	This study
3	fox1_france	Fox	*Vulpes vulpes*	France	KT961030	This study
3	fox2_france	Fox	*Vulpes vulpes*	France	KT961031	This study
3	fox3_france	Fox	*Vulpes vulpes*	France	KT961032	This study
4	canis19	Dog	*Canis lupus familiaris*	Australia	AY493394	[35]
5	canis9	Dog	*Canis lupus familiaris*	USA	AY493395	[35]
6	Sc135	Raccoon dog	*Nyctereutes procyonoides*	Japan	AB821012	[12]
6	Sc108	Dog	*Canislupus familiaris*	Japan	AB821011	[12]
6	Sc34	Raccoon dog	*Nyctereutes procyonoides*	Japan	AB821007	[12]
6	Sc18	Raccoon dog	*Nyctereutes procyonoides*	Japan	AB821004	[12]
6	dog2ch	Dog	*Canis lupus familiaris*	China	KJ499544	[33]
7	dog1ch	Dog	*Canis lupus familiaris*	China	KJ748527	[33]
8	dog3ch	Dog	*Canis lupus familiaris*	China	KJ499545	[33]
9	dog4ch	Dog	*Canis lupus familiaris*	China	KJ748529	[33]
10	dog5ch	Dog	*Canis lupus familiaris*	China	KJ748528	[33]
11	Canis aureus	Jackal	*Canis aureus*	Israel	KP987792	[36]
11	Vulpes	Fox	*Vulpes vulpes*	Israel	KP987794	[36]
11	S42	Human	*Homo sapiens*	France	-^b^	[32]
12	hominis208	Human	*Homo sapiens*	Australia	AY493382	[35]
12	S60	Human	*Homo sapiens*	France	-^c^	[32]
12	1 M	Human	*Homo sapiens*	France	-^c^	[32]
12	2 M	Human	*Homo sapiens*	France	-^c^	[32]
12	9 M	Human	*Homo sapiens*	France	-^c^	[32]
12	4 M	Human	*Homo sapiens*	France	-^c^	[32]
12	5 M	Human	*Homo sapiens*	France	-^c^	[32]
12	7 M	Human	*Homo sapiens*	France	-^c^	[32]
12	S14	Human	*Homo sapiens*	France	-^c^	[32]
12	S45	Human	*Homo sapiens*	France	-^c^	[32]
12	S46	Human	*Homo sapiens*	France	-^c^	[32]
12	S47	Human	*Homo sapiens*	France	-^c^	[32]
12	S48	Human	*Homo sapiens*	France	-^c^	[32]
12	S59	Human	*Homo sapiens*	France	-^c^	[32]
12	S74	Human	*Homo sapiens*	France	-^c^	[32]
13	hominis13	Human	*Homo sapiens*	Australia	AY493383	[35]
14	hominis14	Human	*Homo sapiens*	Australia	AY493384	[35]
15	S32	Human	*Homo sapiens*	France	KR058184	[32]
15	S7	Human	*Homo sapiens*	France	-^d^	[32]
15	S9	Human	*Homo sapiens*	France	-^d^	[32]
15	10 M	Human	*Homo sapiens*	France	-^d^	[32]
15	S12	Human	*Homo sapiens*	France	-^d^	[32]
15	S15	Human	*Homo sapiens*	France	-^d^	[32]
15	S20	Human	*Homo sapiens*	France	-^d^	[32]
15	S21	Human	*Homo sapiens*	France	-^d^	[32]
15	S27	Human	*Homo sapiens*	France	-^d^	[32]
15	S11	Human	*Homo sapiens*	France	-^d^	[32]
15	S25	Human	*Homo sapiens*	France	-^d^	[32]
15	S29	Human	*Homo sapiens*	France	-^d^	[32]
15	S38	Human	*Homo sapiens*	France	-^d^	[32]
15	S40	Human	*Homo sapiens*	France	-^d^	[32]
15	S44	Human	*Homo sapiens*	France	-^d^	[32]
15	S50	Human	*Homo sapiens*	France	-^d^	[32]
15	S51	Human	*Homo sapiens*	France	-^d^	[32]
15	S56	Human	*Homo sapiens*	France	-^d^	[32]
15	S30	Human	*Homo sapiens*	France	-^d^	[32]
15	S34	Human	*Homo sapiens*	France	-^d^	[32]
15	S39	Human	*Homo sapiens*	France	-^d^	[32]
15	S57	Human	*Homo sapiens*	France	-^d^	[32]
15	S8	Human	*Homo sapiens*	France	-^d^	[32]
15	13 M	Human	*Homo sapiens*	France	-^d^	[32]
15	15 M	Human	*Homo sapiens*	France	-^d^	[32]
15	20 M	Human	*Homo sapiens*	France	-^d^	[32]
15	S69	Human	*Homo sapiens*	France	-^d^	[32]
15	S71	Human	*Homo sapiens*	France	-^d^	[32]
16	S58	Human	*Homo sapiens*	France	KR058186	[32]
17	8 M	Human	*Homo sapiens*	France	-^e^	[32]
18	18 M	Human	*Homo sapiens*	France	KR058187	[32]
19	dog1_china	Dog	*Canis lupus familiaris*	China	KT961021	This study
19	dog5_china	Dog	*Canis lupus familiaris*	China	KT961023	This study
20	dog4_china	Dog	*Canis lupus familiaris*	China	KT961028	This study
20	dog2_france	Dog	*Canis lupus familiaris*	IDF/France	KT961024	This study
20	dog_SthAfr	Dog	*Canis lupus familiaris*	South Africa	KT961026	This study
21	dog_thd	Dog	*Canis lupus familiaris*	Thailand	KT961027	This study

^a^This sequence is identical to that of canis22 (AY493393)

^b^This sequence is identical to that of waterbuffalo 37025 (AB779588)

^c^This sequence is identical to that of hominis205 (AY493382)

^d^This sequence is identical to that of S32 (KR058184)

^e^This sequence is identical to that of PIG1 (KR058185)

### DNA extraction and gene amplification

Mite genomic DNA was individually extracted with NucleoSpin Tissue kit, Macherey-Nagel, Germany [26, 27]. A part of cytochrome *c* oxidase subunit 1 (*cox*1) gene was amplified. PCR was carried out in 50 μl and reaction mixture contained 1X PCR buffer, 2.5 mM MgCl_2_, 1 mM of dNTPs, 1.25U DNA polymerase AmpliTaq Gold (Applied Biosystems, Courtaboeuf, France) and 0.25 μM of primer (NavF : 5’-TGATTTTTTGGTCACCCAGAAG-3’; NavR : 5’-TACAGCTCCTATAGATAAAAC-3’) [28]. Amplification conditions were as follows: an initial denaturation step at 94 °C for 5 min, followed by 35 cycles of denaturing at 94 °C for 30s, annealing at 51 °C for 30s, and extending at 72 °C for 40s and a 5 min of final extension at 72 °C.

### Sequence and phylogenetic analyses

The PCR-amplified products of 400 bp were purified and directly sequenced. The *Otodectes cynotis cox1* sequence (KF891933) was retrieved from GenBank. Multiple sequence alignments of nucleotide sequences in this study and sequences available from GenBank (*n* = 81) were generated using MAFFT v.6.951. The dataset was analyzed with Maximum Likelihood using MEGA5 and RAxML-HPC v7.0.4 under General Time-Reversible (GTR + G) model and Bayesian Inference analysis. Support of internal branches was evaluated by non-parametric bootstrapping with 500 replicates. Bayesian Inference analysis was performed with MrBayes v.3.2.1 conducting in two simultaneous runs with four parallel Markov chains (one cold and three heated) for 1 million generations, sampling every 1000 generations and discarding the first 25 % of samples as burn-in. Potential Scale Reduction Factor approached 1.0 and average of split frequencies under 0.01 were used for examining convergence. All trees were visualized using FigTree with *Otodectes cynotis* as outgroup (http://tree.bio.ed.ac.uk/software/figtree). To visualize the relationships between haplotypes, a median joining haplotype network of *cox*1 sequence was constructed using Network v4.6 according to host.

## Results

The sequences of *cox*1 fragment were obtained in mites from nine dogs and three foxes (Table 1). All sequences were deposited [GenBank: KT961021-KT961032]. Other sequences corresponding to 50 mites from humans, raccoon dogs (*Nyctereutes procyonoides*) (*n* = 6), fox (*n* = 1), jackal (*Canis aureus*) (*n* = 1) and domestic dogs (*n* = 11) and from various geographical areas were retrieved from GenBank and from a previous study (Table 1).

All of the successfully sequenced samples were assigned to only one haplotype. In all, 21 haplotypes were observed among mites collected from five different host species, including humans and canids, and nine geographical areas (Table 1). Seven haplotypes were observed among mites collected in humans (H12-H18); two haplotypes were shared with mites collected from canids and human (H3 and H11) and 12 haplotypes (H1-H2, H4-H10, H20-H21) were observed among mites collected from canids.

**Fig. 1:**
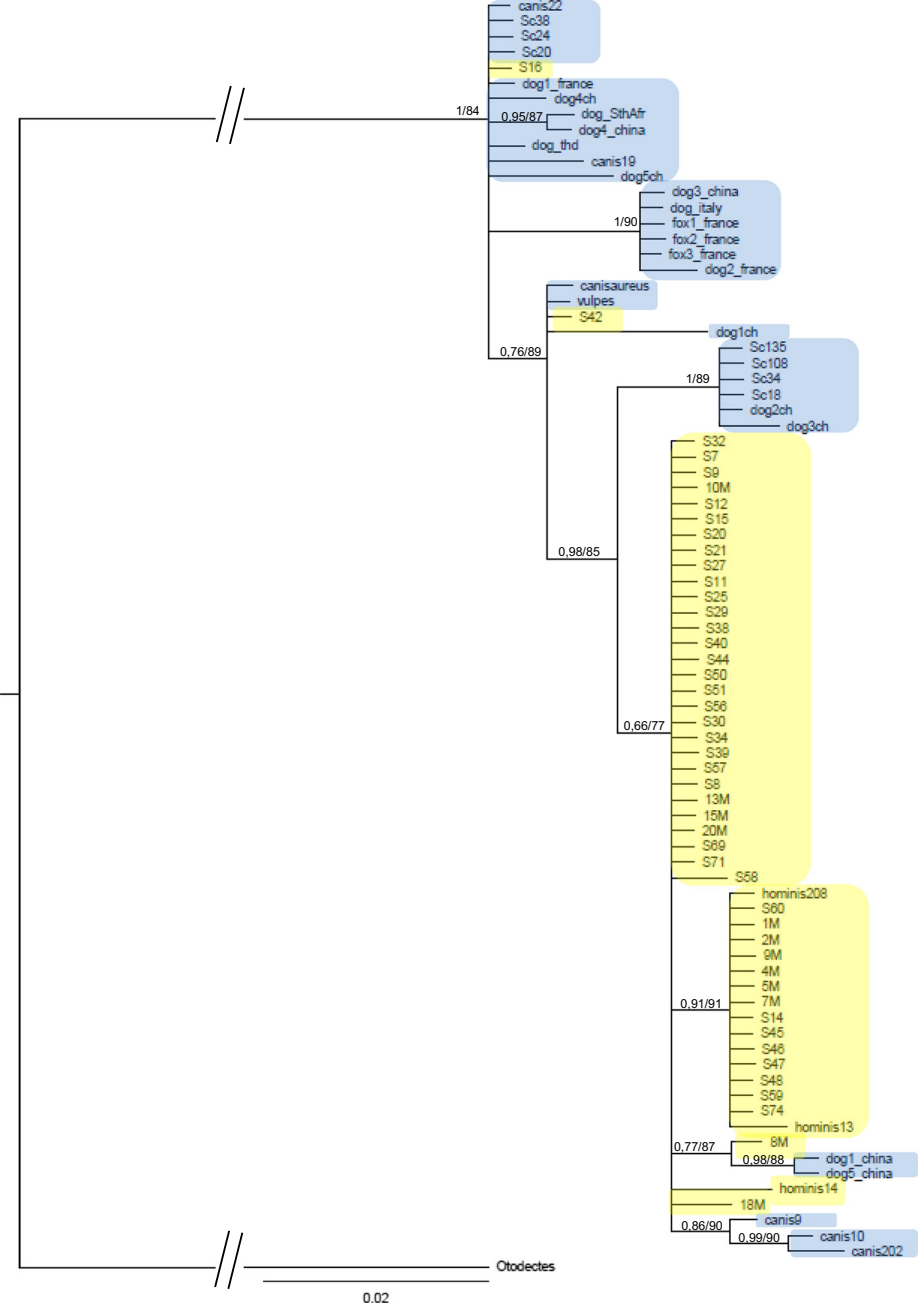
Phylogenetic tree among *Sarcoptes scabiei* from canids and humans. Bootstrap values are indicated above branches, left of the slash for Maximum Likelihood and right of the slash for Bayesian Inference. Tree was rooted with *Otodectes cynotis* (KF891933). Blue shading: mites collected from canids. Yellow shading: mites collected from humans

Sequences from dogs (*n* = 20), raccoon dogs (*n* = 6), foxes (*n* = 4), Jackal (*n* = 1) and humans (*n* = 50) were used to construct the phylogenetic trees based on Maximum Likelihood and Bayesian Inference analyses. They showed similar topologies with few differences in node support values (Fig. 1).

**Fig. 2:**
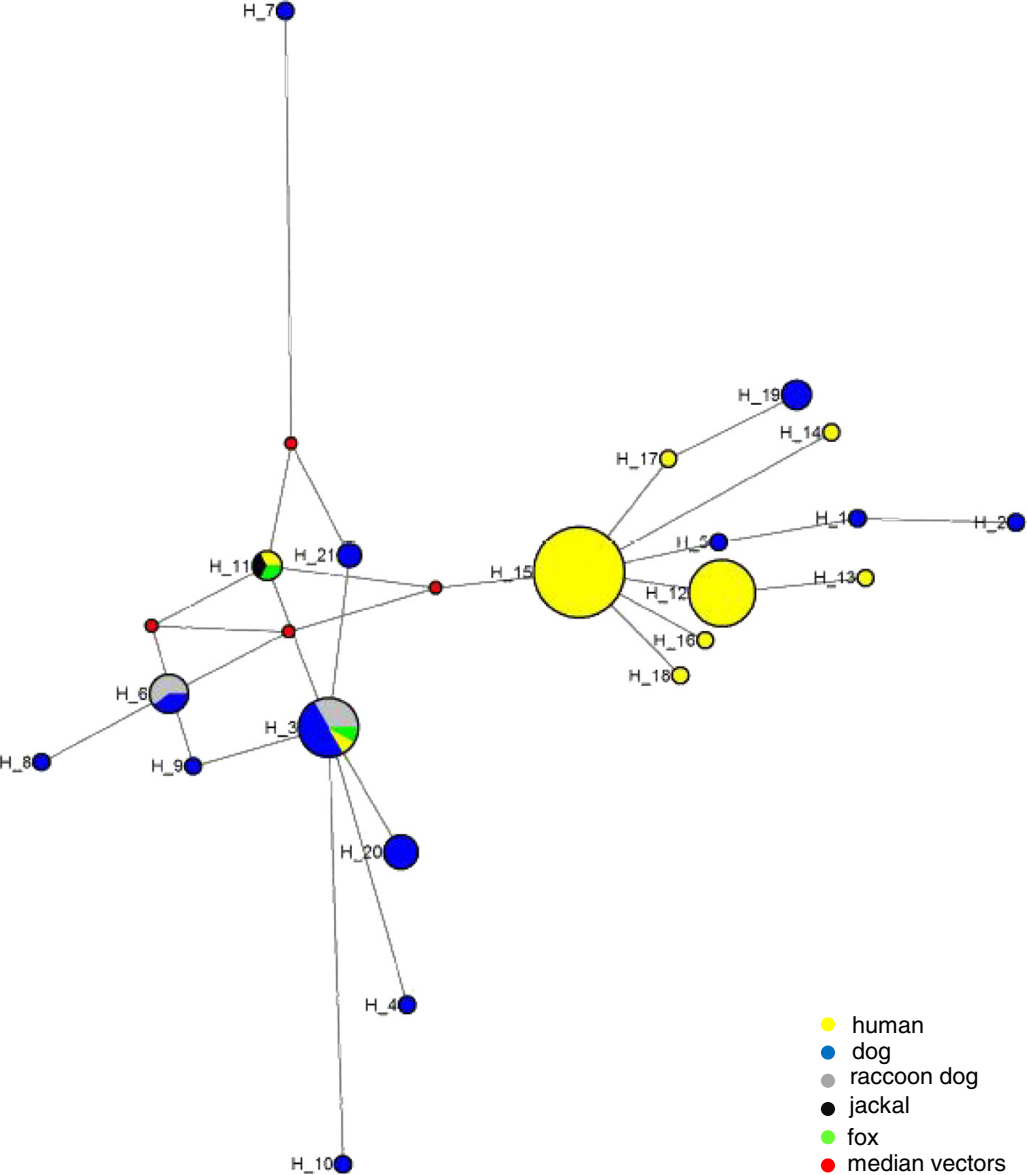
Haplotype map of *Sarcoptes scabiei* from canids and humans inferred under median joining. Size of circles is proportional to haplotype frequency. Median vectors correspond to possibly extant un-sampled sequences or extinct ancestral sequences

The haplotype network showed two distinct populations of mites, a relatively diverse population from dogs and other canids, and a more homogeneous population from humans (Fig. 2). In addition, values of haplotype diversity (Hd) and nucleotide diversity (π) indicated a larger genetic diversity for *S. scabiei* mites collected in dogs than for those collected in humans (Table 2).

**Table 2:** Estimates of genetic diversity of *Sarcoptes scabiei* mites from humans and canids

	No. of sequences	No. of haplotypes	Haplotype diversity (Hd) (± sd)	Nucleotide diversity (π) ± (sd)
Humans	50	9	0.606 (0.056)	0.0022 (0.00041)
Dogs	20	13	0.942 (0.034)	0.011 (0.0012)
Canids (including dogs)	31	14	0.871 (0.046)	0.0087 (0.0011)

## Discussion

The historical hypothesis about the origin of *S. scabiei* in dogs is a transfer of parasites from humans to their domestic dogs. Under this scenario, the population of mites from humans should be basal in the phylogenetic tree. This is not what was observed in the present phylogenetic analyses. Our data were not consistent with a human origin of *S. scabiei* in dogs. On the contrary, our results did not exclude the opposite hypothesis of a host switch from dogs to humans. The haplotype network showed also that, on two occasions, haplotypes from dogs, H19 and H5, H1, H2, seemed to derive from *S. scabiei* mites in humans. Being possibly of canine origin, mites infecting humans may in some occasions return to canine hosts.

The fact that non-human primates are not affected by scabies (or the few times it was described it was considered that this was *via* a human contamination [29]) while the brother genera of *Sarcoptes* (*Otodectes* and *Psoroptes*) infect carnivores or sheep (phylogenetically closer to dogs than human) reinforces the hypothesis of a canine origin of scabies and a host transfer to humans [30].

According to the historical hypothesis, behavioral transmission between humans and dogs occurred when humans domesticated various species of animals at the beginning of agriculture and sedentarization [3]. The origin of the domestic dog is still debated. Recent data indicate that domestic dogs evolved from a group of wolves that came into contact with hunter-gatherers between 18,800 and 32,100 years ago [31]. Those data contradict the historical hypothesis as agriculture was developed later, around 11,500 years ago.

We included all the *cox*1 nucleotide sequences of *S. scabiei* available in GenBank that were from canids and from all human mites sharing the same clade as canid mites in published phylogenetic studies (Table 1). *Cox*1 gene, including a very high number of polymorphisms, was found to be valid and best suited for this type of phylogenetic analysis according to previous studies on the same topic [32, 33].

Mites of human origin were collected in only two countries, mostly in France. It does not necessarily mean that patients acquired their mites in France. Indeed, various ethnic communities are represented among the outpatients that visit our departments (about one third are immigrants) and it is likely that a not-insignificant number of cases of scabies were acquired abroad. However, we cannot formally exclude that a sampling bias could have led us to underestimate the diversity of *cox*1 in human mites.

Host switching promotes *S. scabiei* diversification and reflects the exceptional dissemination potential of these mites among various species of mammals. Scabies spreading in wild populations may occur on an epidemic mode and may be devastating for naive populations because of the lack of immunity [34]. It may be underlined that transmission between dogs and humans still occurs. In a recent study, Zhao et al., using *cox*1 for phylogenetic analysis, reported that mites from dogs in China, Australia and USA clustered with mites collected from Australian people [33]. Those authors concluded that humans could be infected with mites from dogs. The present data and our previous results on this point are in agreement with those authors [32]. Those authors also conclude that geographical isolation was observed between human mites. The aim of our study was not to explore a possible geographic effect on *Sarcoptes* evolution but to present documented data on the possibility that humans are the initial source of canine mange. We agree that geographic clustering occurs in human *Sarcoptes* evolution [32] but this seems not to be the case for canid *Sarcoptes*. Indeed, our phylogenetic tree argues against any geographical effect on canid *Sarcoptes* evolution because most of the clades are made of taxa from different locations (for example a clade shows that foxes and dogs from France clustered with dogs from China in Fig. 1). Nevertheless, other studies including more *S. scabiei* mites from canids originating from different locations are needed to answer this question.

Two mites collected in humans, S16 and S42, belonging to haplotypes shared by mites from humans and canids, clustered with mites collected in canids in the present study (Fig. 1 and Table 1). In addition, some other haplotypes may be shared by different hosts, as shown in this study and in other works [20, 32]. Thus, the historical hypothesis of the “high degree of host- specificity and low degree of cross-infectivity of *S. scabiei*” [10] is challenged.

## Conclusions

Phylogenetic relatedness may have an impact in terms of epidemiological control strategy. Our results and other recent studies suggest to re-evaluate the level of transmission between humans and animals and between domestic and wild animals [16, 30]. In particular, it may be useful to know the proportion of human scabies contracted from infected dogs and also whether cases of sarcoptic mange in dogs may be due to mites from humans.

Control programs for human scabies should consider concomitant programs for mange in dogs to optimize efficacy. In addition, the existence of some degree of gene exchange between host-associated populations should be considered for the surveillance of the emergence and diffusion of insecticide resistance.
